# In death there is life: perceptions of the university community regarding body donation for educational purposes in the United Arab Emirates

**DOI:** 10.1016/j.heliyon.2021.e07650

**Published:** 2021-07-22

**Authors:** Nerissa Naidoo, Ghadah A. Al-Sharif, Raeesa Khan, Aida Azar, Amar Omer

**Affiliations:** a College of Medicine, Mohammed Bin Rashid University, Dubai Healthcare City, Dubai, United Arab Emirates; b Hamdan Bin Mohammed College of Dental Medicine, Mohammed Bin Rashid University, Dubai Healthcare City, Dubai, United Arab Emirates

**Keywords:** Body, Body donation, Research and education, Anatomy, Perceptions

## Abstract

Human body dissection is the traditional instructional method for anatomy education worldwide, providing a kinaesthetic learning experience that is often challenging to achieve with other teaching techniques. However, due to lack of body donation programs in Middle Eastern medical schools, dead bodies are imported from abroad.

Since literature suggests that the body shortage is influenced by reluctance to donate one's body, this study aimed to determine the perceptions of faculty, staff, and students regarding body donation for educational purposes at a new Dubai-based medical school.

An online dually translated questionnaire was administered to the target population (322), of which 150 participants representative of faculty, staff, and students, responded.

Although 111 (74.0 %) of participants considered body donation to be appropriate for educational and research purposes, only 44 (29.3 %) of participants expressed willingness to donate their bodies. Reluctance to donate 106 (70.7 %) appeared to be mostly influenced by religion, psychological barrier, and familial reasons. The emergence of four themes (i.e., resource, barrier, humanitarian, and awareness) and the identification of a potential donor group within the group that was willing to donate provided insight into the level of awareness within the university community. Furthermore, such findings may assist to establish future body donation programs and strategize recruitment approaches, especially when there is an ensuing dearth of anatomical donations.

## 1 Introduction

The Latin phrase “Taceant colloquia. Effugiat risus. Hic locus est ubi mors gaudet succurrere vitae’’, which translates to “Let conversation cease. Let laughter flee. This is the place where death delights to help the living”, adorns the entrances of many mortuaries and anatomy dissection halls around the world [1]. While there is much speculation around the literal meaning of this phrase, it is undeniable that it conveys the essence of the respect and honour that should ensue as one sets foot into the mortuary or dissection hall. It also emphasizes the value and role of a dead body in the educational journey and future professional practice of an undergraduate medical student [2, 3]. Not only does the traditional hands-on approach of dissecting the body allow the student to simultaneously learn the gross anatomical structure while using one's physiological senses, but it also conveys medical ethics and best practices, and helps to cultivate humanistic values and core professional competencies, thereby instilling the “hidden curriculum” of life [4, 5]. Hence, human body dissection in the discipline of anatomy has remained as the primary method of anatomy instruction for centuries and is often deemed to be a fundamental component of the pre-clinical phase in the Undergraduate Medical Education (UME) curriculum [3, 6]. However, hidden behind this ceremonial learning journey, lies the tale of body procurement [7].

### 1.1 Historical perspective on the procurement of human bodies

Historically, the procurement of bodies for anatomization dates to the 3rd century BC during which the Greek physician, Herophilus of Chalcedon – known as the Father of Anatomy, popularized human body dissection in Alexandria [8, 9]. However, with the fall of the Roman Empire and the emergence of Christianity in the Middle Ages, came the prohibition of human body dissection as it was considered to be blasphemous [9, 10]. Instead, scientific quest in medicine was greatly discouraged and physicians were expected to emulate the works of Aristotle and Galen [7]. Although human body dissection began its revival in the Late Middle Ages through bi-annual public dissection events held by the University of Bologna and the issuance of a papal decree calling for the autopsies of plague victims, the Renaissance was ultimately responsible for the re-introduction of human body dissection as this period saw human anatomy through the unique lens of art and medical science [9, 11]. This sparked interest in the artistic and scientific aspects of human anatomy, with the resultant demanding increase in dead bodies causing a shortage in supply [7]. The dearth of bodies in these fields was initially sustained through physician-recommended post-mortem and provision of unclaimed bodies by charitable hospitals [9, 12]. Yet, with time as anatomy flourished and new medical schools were established, these sources could not meet the growing need of human bodies for dissection [9, 13]. In the centuries that followed, the unspoken hunger for human bodies was the driving force behind body procurement, with many resorting to various malpractices such grave-robbing, vivisection, and “Burking” [8, 14, 15, 16, 17]. In fact, some medical and criminal justice systems colluded to manipulate the time and mode of capital punishment to match the dissection needs [12]. Consequently, the United States of America (USA) and the United Kingdom enacted common laws and acts (i.e., Murder Act, Massachusetts Anatomical Act of 1831, Anatomy Act of 1832, Maine's Anatomy Act of 1869) that legalized the immediate procurement of unclaimed bodies from state institutions to local medical schools, thereby protecting the lives of the living and ending the malicious malpractices [8, 9, 18]. With these laws in place, societal perceptions about death and dissection were positively influenced as people became more open to the idea of donating one's body to science, with a Maryland horse dealer (Thomas Orne) being the first to pledge his body in 1899 [18]. As the development of transplant surgery also gained momentum during this time, the Uniform Anatomy Gift Act of 1968 was later passed in the USA and mandated that donating one's body to science was based purely on personal choice and volunteerism [9, 16, 18].

### 1.2 The current global situation of anatomy education

With the introduction of the E-learning era and global transformation of the social milieu over the last decade, the delivery of anatomy education has also evolved, reducing human body dissection to computer-assisted instruction, medical imaging and plastic models [2, 3, 6]. As a result, the discipline of anatomy has seen a decline in the number of skilled pure anatomists and an influx of surgical specialists, with the educational design adopting a strong clinical outlook, rather than the basic medical science outlook initially required in the foundational phases [2].

In addition to these technological advances, the procurement of dead bodies from medico-legal (i.e., unclaimed bodies) or donated sources has also posed many challenges as it continues to be bound by red tape and regulation [2, 19]. Moreover, the existence of numerous geographical differences in ethical and legal frameworks that govern body donation for teaching and scientific research has emphasized the need for standardized guidelines of good practice [20, 21]. Consequently, in August 2014, the International Federation of Associations of Anatomists (IFAA) issued the “Recommendations of good practice for the donation and study of human bodies and tissue for anatomical examination” to provide countries facing challenges with approved international guidelines to establish body donation programs in their respective institutions, as well as grant potential donors absolute confidence in their decision to donate for the advancement of science and education [20, 22]. With these approved guidelines at the forefront, many tertiary institutions then attempted to address the body shortage by implementing body donation programs. However, this was met with concerns pertaining to the socio-demographic factors of the country and method of disposal – the latter hinging on the laws and legislations by which a particular country abides [23]. Nevertheless, Strkalj and Pather [24] provided a plausible means to address this by “humanizing anatomy” through commemoration and memorial services that pay homage to the donors and their families and express appreciation for the gift bestowed upon the medical education community.

### 1.3 What is the situation in the Middle East?

Similarly, in the Middle East (ME), the scarcity of bodies has been a prominent issue, with an Omani study briefly addressing it in 1994 [25]. The growing establishment of new medical schools in the Kingdom of Saudi Arabia has also increased the demand for bodies in the UME and postgraduate surgical curricula [26]. While the majority of these ME institutions source this material from body-exporting markets world-wide, this involves many tedious technicalities to ensure all ethical and logistical concerns are resolved [26, 27]. Indeed, this also sheds light on a larger ethical issue encircling body broker companies as many donor families have reported that they did not realize that pledging one's body also entailed dismemberment of parts and exportation of it to other regions of the globe for international use as this was not outlined explicitly in consent forms. Moreover, certain body broker companies have come under fire for the exportation of infected bodies, which the Center for Disease Control and Prevention (CDC) declared could pose detrimental effects on healthcare workers [28].

On another note, the paucity in available human body specimens in the ME is also attributed to the absence of whole-body donation programs. Interestingly, organ donation has gained much popularity and acceptance in the ME, with the United Arab Emirates (UAE) passing a Federal Decree Law on the Regulation of Human Organs and Tissue Transplantation in 2016 [29]. However, the concept of body donation for education and research purposes is yet to be considered, with reluctance to donate one's body speculated to be influenced by personal perceptions and respective socio-demographics factors [3, 6, 19, 30, 31].

Therefore, through a student research project, this study aimed to determine the perceptions of faculty, staff, and students regarding body donation for research and education in a new Dubai-based medical school.

## 2 Methods

### 2.1 Study design

This was a cross-sectional study conducted at Mohammed Bin Rashid University (MBRU), Dubai, UAE in the College of Medicine, and the Hamdan Bin Mohammed College of Dentistry.

### 2.2 Study population

As MBRU is a new university, the complete coverage population of students, faculty, and staff (N = 322) was considered to be eligible for this research study, however only 47 % of the target population responded. Since this study included the whole population and not a sample of it, the research team deduced that a response rate of at least 50 % would be considered excellent a priori to the dissemination of the online questionnaire [32].

### 2.3 Study material

The questionnaire, which consisted of 13 question items reflective of the multiple-choice and open-ended varieties, was defined by socio-demographic information, and perceptions and attitudes towards body donation for education and research.

### 2.4 Validity and reliability

The questionnaire was initially drafted by two experts in the field to verify its validity (i.e., expert validity). Once a consensus was reached, the questionnaire was disseminated through a pilot study prior to the start of the actual study. Five students participated in the pilot study. All five respondents were able to answer all the questions, confirming reliability of the questionnaire. Based on the constructive feedback received from the five respondents, the questionnaire was revised accordingly, thus ensuring validity of it.

As per the guidelines of the MBRU Institutional Review Board (IRB), the questionnaire was translated into the Arabic language. This was done to ensure that all native Arabic speakers were also able to understand the content, thereby providing both native English and Arabic speakers with equal opportunity to express their perceptions and thoughts with ease. The internal consistency (i.e., reliability) of the questionnaire was then evaluated by inviting five bilinguals (proficient in both English and Arabic language) to respond to both the English and Arabic versions of the questionnaire. The results revealed 100 % correlation between the translated and original questionnaires.

**Figure 1:**
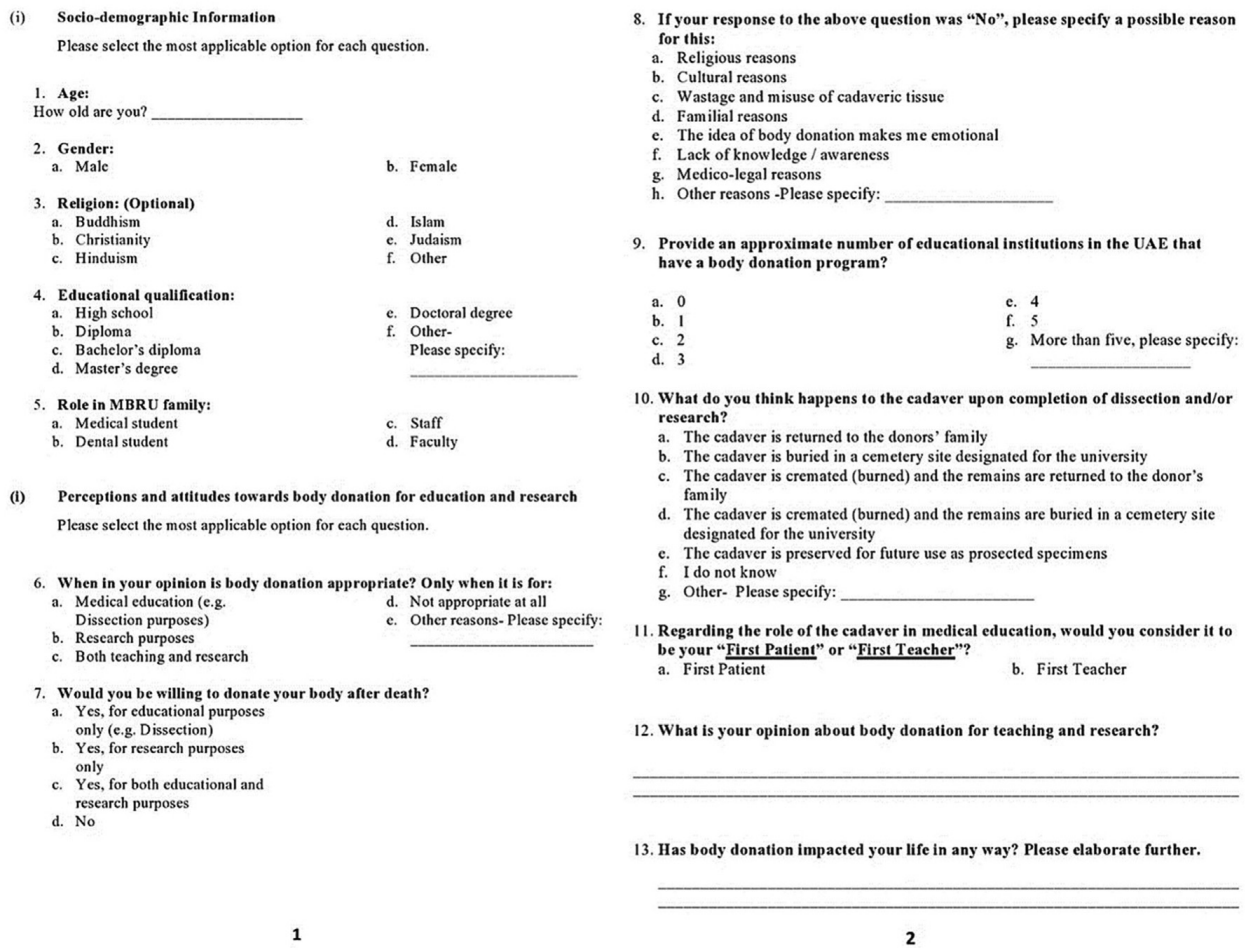
Standardized English questionnaire.

**Figure 2:**
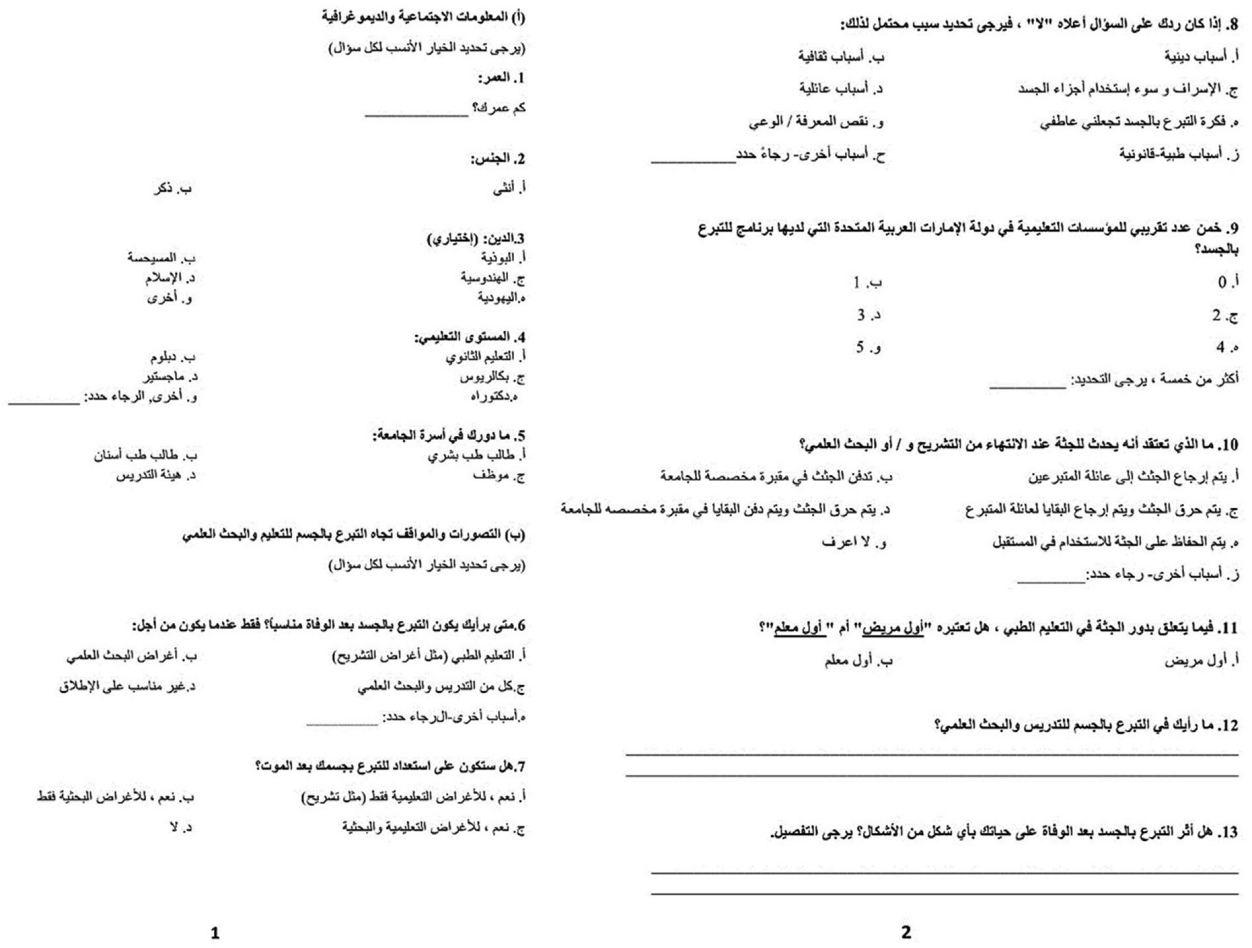
Arabic version of questionnaire.

The validated English and Arabic versions of the questionnaire (Figures 1 and 2) were then administered to all students, staff, and faculty via an email link to the online Google forms 2018 platform. Individuals initially confirmed their participation in this study through an e-informed consent form, which stated the study rationale and indicated that participation in study was completely voluntary. The questionnaire email link was shared twice with the entire study population, with a response rate of 47 % (150/322) eventually achieved. The data emanating from the questionnaire was collected via a collation tool on the Google forms platform.

### 2.5 Ethical approval

Ethical approval was obtained from the MBRU-IRB (Application number MBRU-IRB-2018-004).

### 2.6 Data analysis

#### 2.6.1 Quantitative analysis

Quantitative analysis was conducted by using the Pearson Chi-Square Test (IBM SPSS Statistics for Windows, Version 26.0), through which the dependency between the categorical variables was determined. A P-value <0.05 was considered to be statistically significant.

#### 2.6.2 Cluster analysis

A cluster analysis was conducted to identify the existence of a potential body donor group within the participant population; hence the optimal number of clusters was pre-defined as two [33, 34]. The cluster membership for each case was determined by executing the K-means algorithm which then divided the data into clusters. The Pearson Chi-Square test was performed to compare the homogeneity of the selected attributes between the two clusters (IBM SPSS Statistics for Windows, Version 26.0).

#### 2.6.3 Qualitative analysis

The participants’ responses to the two open questions in the category regarding perceptions and attitudes towards body donation for education and research was qualitatively explored using thematic analysis. These responses were critically reviewed and analysed by two independent investigators (GA and NN) to generate emergent themes [35].

## 3 Results

### 3.1 Quantitative analysis

#### 3.1.1 Socio-demographic distribution of the population

In this study, a total of 150 participants responded to the questionnaire from a target population of 322 individuals. All participants of the study group were above the age of seventeen, with a mean age (standard deviation) of 25 (11.4) years. Table 1 shows that three-quarters of participants were females 108 (72.0 %), while one-third of the study population was represented by males 42 (28.0 %). The main population groups were students 112 (74.7 %), followed by staff 22 (14.7 %) and faculty 16 (10.7 %). The majority of participants had completed secondary education 98 (72.1 %), with two-fifths 38 (28.0 %) of the study population holding a graduate or post-graduate certification. Most participants were of the Islamic faith 130 (86.7 %), while the other religious groups were a minority, consisting mainly of Christianity 11 (7.3 %) and Hinduism 6 (4.0 %).

**Table 1:** Socio-demographic distribution of the population.

Socio-demographic factors		All Participants (N = 150) Number (%)
Gender	Male	42 (28.0)
	Female	108 (72.0)
Age (years)	(Mean ± Standard deviation)	25 ± 11
Role of participant	Faculty	16 (10.7)
	Staff	22 (14.7)
	Student	112 (74.7)
Level of education	Secondary education	98 (72.1)
	Bachelor's degree	22 (16.2)
	Post-graduate certification	16 (11.8)
Religion	Muslims	130 (86.7)
	Others	20 (13.3)

#### 3.1.2 Appropriateness of body donation

Table 2 shows that three-quarters of the participant population 111 (74 %) considered body donation to be appropriate for both educational and research purposes. The remaining participants expressed that it is appropriate solely for medical education 12 (8.0 %) or only for research purposes 6 (4.0 %). In addition, organ donation 9 (6.0 %) was predominantly cited in the “Other” category as a factor influencing appropriateness. As students comprised of three-quarters of the participant population, a separate analysis was also conducted for this group. The data revealed no heterogeneity in the outcomes; hence the inclusion of staff and faculty did not skew the data, and the total population was considered in the analysis. Table 2 reveals that no statistical significance (P > 0.05) exists between the total population and the student sample regarding the appropriateness of body donation.

**Table 2:** Frequency distribution of appropriateness of body donation.

Opinion of body donation	Number (%)		P-value
	All Participants^1^ (N = 150)	Students^2^ (N = 112)	
Both educational and research purposes	111 (74.0)	87 (77.7)	0.49
Only for medical education (such as dissection purposes)	12 (8.0)	9 (8.0)	1.00
Only for research purposes	6 (4.0)	2 (1.8)	0.31
Other (such as organ donation)	9 (6.0)	4 (3.6)	0.78

1 Data was analysed for all participants (students, staff and faculty).

2 75 % of the study population comprised of students, hence a separate analysis was performed for the student population.

#### 3.1.3 Willingness to donate

Forty-four (29.5 %) participants were willing to donate, with both education and research 31 (20.8 %) specified as the main purpose (Table 3). On the other hand, 106 (71 %) participants of the study population were reluctant to donate their bodies for educational and/or research purposes (Table 3). Reluctancy to donate was largely influenced by religious reasons 38 (43.2 %), followed by perceptions shaped by one's emotional state 23 (26.1 %) and familial reasons 10 (11.4) (Table 3). Interestingly, the additional analysis conducted on the student population revealed no statistical significance (P > 0.05) between the total study and student populations for willingness or reluctance to donate one's body.

**Table 3:** Frequency distribution of willingness or reluctance to donate one's body for educational and research purposes.

Willingness or reluctance to donate		Number (%)		P-value
		All Participants^1^ (N = 150)	Students^2^ (N = 112)	
Willing N = 44 (29.3 %)	Educational purposes	4 (2.7)	4 (3.6)	0.68
	Research purposes	9 (6.0)	5 (4.5)	0.60
	Education and research purposes	31 (20.8)	20 (18.0)	0.57
Reluctant N = 106 (70.7 %)	Religious reasons	38 (43.2)	28 (41.2)	0.75
	Cultural reasons	5 (5.7)	5 (7.4)	0.58
	Wastage and misuse of cadaveric tissue	6 (6.8)	6 (8.8)	0.55
	Familial reasons	10 (11.4)	9 (13.2)	0.66
	Idea of body donation makes me emotional	23 (26.1)	17 (25.0)	0.84
	Lack of knowledge/awareness	5 (5.7)	2 (2.9)	0.28
	Medico-legal reasons	1 (1.1)	1 (1.5)	0.77

1 Data was analysed for all participants (students, staff and faculty).

2 75 % of the study population comprised of students, hence a separate analysis was performed for the student population.

#### 3.1.4 Correlation between socio-demographic factors and willingness to donate

Table 4 highlights the socio-demographic characteristics of the study population who were willing to donate their bodies (N = 44). Since the mean age of participants was 25 years, this was used as a cut-off value to divide the analysis into two groups, namely, those younger than twenty-five (≤25), and those older than twenty-five years (>25). However, no evidence of association nor dependency was established between willingness to donate (P > 0.05) and age (Table 4). On the other hand, the other socio-demographic factors (gender, role of participant, level of education, and religion) were found to be associated with the willingness to donate (P < 0.05).

**Table 4:** Correlation between socio-demographic factors and willingness to donate.

Socio-demographic factors		Willingness to Donate (N = 44)	
		Number (%)	P-value
Gender	Male	18 (40.9)	0.029∗
	Female	26 (59.1)	
Age (years)	<25	27 (64.3)	0.348
	≥25	15 (35.7)	
Role of participant	Faculty	11 (25.0)	0.001∗
	Staff	4 (9.1)	
	Student	29 (65.9)	
Level of Education	Secondary education	26 (61.9)	0.014∗
	Bachelor's degree	6 (14.3)	
	Post-graduate certification	10 (23.8)	
Religion	Muslim	31 (70.5)	<0.001∗
	Others	13 (29.5)	

∗ Statistically significant P-values (P < 0.05).

#### 3.1.5 Participants’ perceptions: body donation programs, fate, and role of donated body

According to Table 5, one-third of the study population 52 (34.7 %) believed that no institutes in the UAE have body donation programs. It was also noted that almost two-fifths of the study population 54 (36.7 %) were unaware about the fate of the donated body. Incidentally, when asked about the role of the donated body in medical education, the majority of participants 104 (69.3 %) considered it to be their “first teacher”. When the data was analysed separately for the student population, no statistical significance (P > 0.05) was recorded between the total population and student sample regarding the above-mentioned aspects related to the participants’ perceptions.

**Table 5:** Participants’ perceptions regarding the number of institutes in the United Arab Emirates with body donation programs, the fate of the donated body after use for educational and research purposes, and the role of the donated body.

Participants' Perception		Number (%)		P-value
		All Participants^1^ (N = 150)	Students^2^ (N = 112)	
Number of institutes in the United Arab Emirates with body donation programs	None	52 (34.7)	31 (27.7)	0.23
	1	17 (11.3)	12 (10.7)	0.88
	2	29 (19.3)	28 (25.0)	0.27
	3	19 (12.7)	15 (13.4)	0.87
	4	8 (5.3)	8 (7.1)	0.55
	I do not know	25 (16.7)	18 (16.1)	0.90
Fate of the donated body after use for education and research	The cadaver is returned to the donors' family	15 (10.0)	13 (11.6)	0.68
	The cadaver is buried in a cemetery site designated for the university	15 (10.0)	8 (7.1)	0.41
	The cadaver is cremated, and the remains are returned to the donor's family	27 (18.0)	22 (19.6)	0.74
	The cadaver is cremated, and the remains are buried in a cemetery site designated for the university	10 (6.7)	6 (5.4)	0.66
	The cadaver is preserved for future use as prosected specimens	22 (14.7)	20 (17.9)	0.49
	I do not know	54 (36.7)	40 (35.7)	0.87
	Other	7 (4.7)	3 (2.7)	0.41
Role of the donated body as perceived by the participant	First Teacher	104 (69.3)	77 (68.8)	0.93
	First Patient	46 (30.7)	35 (31.2)	0.93

1 Data was analysed for all participants (students, staff and faculty).

2 75 % of the study population comprised of students, hence a separate analysis was performed for the student population.

### 3.2 Cluster analysis

Based on the associations established between socio-demographic factors and the willingness to donate (Table 4), two clusters were selected accordingly (potential donor and reluctant group). Table 6 shows statistically significant differences (P < 0.05) in the distribution of the four socio-demographic characteristics (i.e., gender, religion, educational level, and role of the participant) between the two clusters. This revealed that a dependency exists between both clusters and the different socio-demographic characteristics.

**Table 6:** Comparison of two clusters according to selected attributes.

Selected Attributes		Number (%)		P-value
		Reluctant Group (N = 108)	Potential Donor Group (N = 28)	
Gender	Male	24 (22.2)	15 (53.6)	0.002∗
	Female	84 (77.8)	13 (46.4)	
Religion	Muslim	99 (91.7)	19 (67.9)	0.003∗
	Others	9 (8.3)	9 (32.1))	
Level of Education	Secondary	96 (88.9)	2 (7.1)	<0.001∗
	Bachelor's	12 (11.1)	10 (35.7)	
	Post-graduate	0 (0.0)	16 (57.1)	
Role of participant	Faculty	108 (100)	0 (0.0)	<0.001∗
	Staff	0 (0.0)	14 (50.0)	
	Student	0 (0.0)	14 (50.0)	

∗ Statistically significant P-values (P < 0.05).

### 3.3 Qualitative analysis

**Figure 3:**
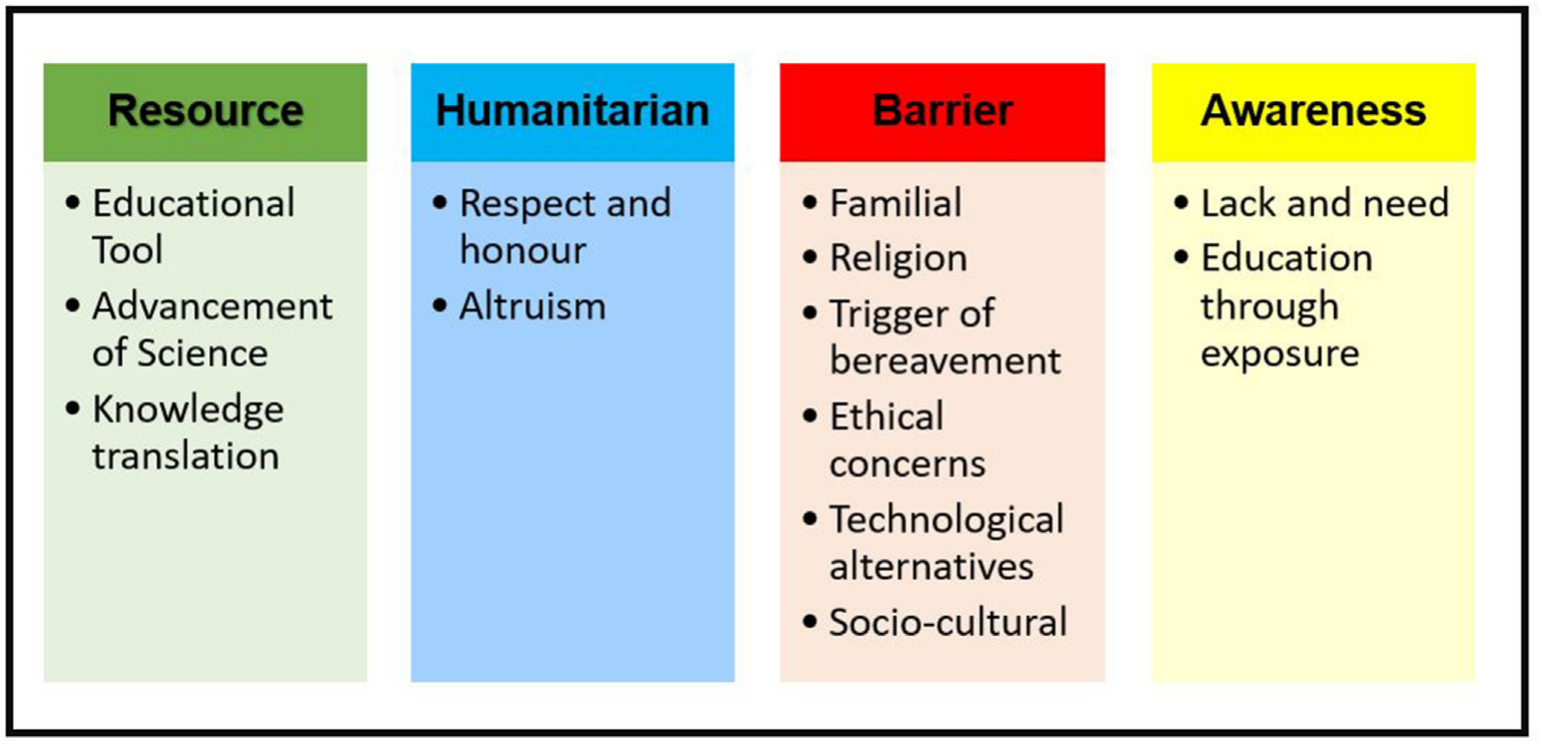
Thematic Framework summarising participants' responses about body donation for educational and research purposes and the impact (direct/indirect) of it on their lives. Legend. From the qualitative analysis, four themes: Resource (green), Barrier (red), Humanitarian (blue), Awareness (yellow) and corresponding sub-themes were generated. The colour distinctions (green and blue) reflect positive connotations towards body donation for research and educational purposes. On the other hand, negative connotations are represented by the red colour. The colour yellow was selected as it provides a way to address positive and negative connotations.

The participants’ responses, emanating from the two open questions regarding perceptions and attitudes towards body donation for education and research, resulted in four colour-coded emergent themes: Resource, Humanitarian, Barrier, and Awareness (Figure 3).

#### 3.3.1 Resource

This theme reflected the participants' perceived value of body donation in medical education and research. Three sub-themes (i.e., educational tool, advancement of science and knowledge translation), that were also noted to emerge from this theme, are highlighted by the participants’ responses provided below (Figure 3):

Response 1: *“Useful and important for the teaching of medical students. Allows the student to understand that human bodies vary and are not exact replicas of each other (from the inside of course) - so just like we differ on the outside we do also on the inside.”*

Response 2: *“I think it is a great way to test the knowledge of the medical students for teaching purposes. As for research purposes, it would benefit the discovery of new medical findings. Therefore, in both ways, it would provide a path for knowledge.”*

Response 3: *“It was the best way to visualize the anatomy of the human being. Relating what was being taught in the lecture to real life.”*

#### 3.3.2 Humanitarian

This theme shed light on the humanitarian elements associated with the act of body donation for education and research purposes. As outlined in the quotes below, participants alluded to the sub-themes of respect and honour, and altruism:

Response 1: *“Body donation has enabled me to develop an immense appreciation for science and the individuals who have selflessly donated their remains to teach the youth of today.”*

Response 2: *“That someone I never knew would choose to be dissected for my educational benefit was surprising and humbling. I respected that human body more than some living people.”*

Response 3: *“I have high respect and regard for the individuals that donated their body as it’s a very selfless and giving act in the aim of bettering the future generations' education and healthcare.”*

#### 3.3.3 Barrier

The theme “Barrier” emerged as an umbrella theme encompassing the following sub-categories: familial, religion, trigger of bereavement, ethical concerns, technological alternatives, and socio-cultural. Participants expressed these sub-themes in many instances through the excerpts listed below:

Response 1: *“My family for instance thinks that it is absurd to donate organs, let alone body donating.”*

Response 2: *“We still do not have the culture of body donation for teaching and research purposes in the UAE. The advancements in technology have mimicked in a very accurate way the anatomy of a cadaver. This can help replace body donation programs.”*

Response 3: *“I think it's a great way to study medicine, but our religion banned it so we can't donate our bodies.”*

Response 4: *“After seeing cadavers for the first-time last year, I felt very emotional. I also felt uncomfortable on many occasions, as it didn't seem right to me that we were opening up a human body, and holding a human heart of a person that lived, a person who once had a family and house, it just didn't feel right to me.”*

Response 5: *“It's unethical.”*

#### 3.3.4 Awareness

In this theme, participants’ responses reflected the lack of awareness regarding body donation for educational and research purposes, the need for awareness and how exposure to it educates individuals about it:

Response 1: *“I have never read about body donations.”*

Response 2: *“It is important in this field but there is still a lot of misunderstanding and lack of transparency circulating the act of donation.”*

Response 3: *“It made me appreciate the concept of body donation more, so I started thinking more about it, and now I am actually interested in it to the point of actually doing it.”*

## 4 Discussion

In the medical and allied health science fields, perceptions about body donation for educational and research purposes are particularly valuable as these communities require the specimens of dead bodies for teaching and study, all of which translate into improved treatment strategies in clinical practice [6]. Hence, it is necessary to consider various factors when the question of body donation arises in this context.

### 4.1 Quantitative analysis

#### 4.1.1 Socio-demographic distribution of the population

In the present study, half of the target population responded to the questionnaire, which, according to Tabachnick and Fidell [36] is a sufficient sample size. The socio-demographic distribution of the sample was also reflective of the rich cultural and nationality diversity at MBRU. In view of the large female participant population, similar trends were also observed in previous studies conducted at MBRU, indicating that the sustainability of the demographics in this study was indeed representative of the total institutional population [37, 38]. Moreover, other universities revealed a similar trend in gender distribution [39].

#### 4.1.2 Appropriateness of body donation

Since anatomists around the globe support the notion that they are living in a transitional time in which post-mortem body donation plays an integral role in sustaining the discipline of anatomy, awareness about how individuals within the medical and academic communities perceive body donation for educational and research purposes, may provide cues on the future of the activating learning experience gained in the dissection room [40]. Interestingly, the majority of participants in this study were of the opinion that body donation is appropriate for both educational and research purposes. Although slightly lower, this positive attitude was in agreeance with that reported by Saha et al. [19]. According to Victor et al. [3], participants who support the act of body donation for both research and educational purposes, tend to identify the role of whole-body dissection in their academic trajectory and understand that it is necessary to grasp the complexity of the human body. On the other hand, the minority group of participants who stated that body donation is appropriate only for medical education (such as dissection), accurately reflected the perspective of participants in a previous study [6].

It was also noted that within the group opining “appropriateness”, a few participants were only in favour of organ donation, an outlook that Kostorrizos et al. [41] ascribed to the fact that body donation for scientific purposes may be deemed less useful than organ donation as the latter could be seen as an altruistic act that directly and immediately decreases the scale of suffering of many patients. While this was similar to the perceptions conveyed by Saha et al. [19] and Quiroga-Garza et al. [2], the response rate was comparably lower. As the earlier public awareness study of Janahi et al. [42] determined that the UAE population were moderately well-informed and motivated about transplantation and organ donation, this may be an influential factor behind organ donation. Familiarity with organ donation is also likely to arise from the known establishment of organ donation programs in the UAE and the ME [43]. Moreover, post-mortem body donation programs do not currently exist in the ME. Surprisingly, participants who were of the belief that body donation is inappropriate for any such purpose in the academic setting, were predominantly medical students who were in the clerkship phase and had already participated in dissection during their anatomy course or were in the pre-clerkship phase and were yet to undertake the course, which is a compulsory component of the anatomy curriculum at MBRU. In contrast to the smell of the dissection room and the touch and fear of the dead body highlighted by Getachew [44], Saha et al. [19] found that the mishandling and the degraded condition of the dead body were also responsible for the negative opinion of students. However, attending an initiation ceremony to commemorate the life once lived by the dead body donor in the presence of the donor family has been shown to reduce students’ negative emotions and change their attitude towards death [45].

#### 4.1.3 Willingness to donate

Although 86 % of participants found body donation to be appropriate for educational and/or research purposes, there was an overall reluctance to donate one's body, thereby corroborating the findings of previous studies [2, 6, 31, 46, 47]. Given the nature of the population group at hand, this sheds lights on the need for awareness and educational campaigns as students and faculty are the vehicles of encouragement and information for family and patients [41].

Religion was cited as the main reason behind reluctance to donate one's body in this study. It is not uncommon that this response was met with reservation as Daar [48] stated that the barrier of religion is more often imagined than real. Similarly, other reports have also identified religion as the predominant factor against willingness to donate [19, 41, 49]. Nevertheless, while religion remains a topic of much discussion, all religions encourage and support whole body and/or organ donation for the benefit of the world, with the ultimate decision to donate left to the individual [41].

It is worth noting that in one-quarter of the sample population, reluctance to donate stemmed from a psychological barrier. Victor et al. [3] associated this perception to having one's own body dissected. Since this view was chiefly expressed by MBRU medical students, it possibly arose from their prior experience in the dissection hall and may be perceived as discouraging to the general public [6, 41, 50]. Likewise, in this case, holding a memorial ceremony may help to humanize the practice of body dissection for educational use, thereby displaying appreciation for this selfless act [51].

The proportion of participants willing to donate their bodies was substantially lower in this study as compared to other studies [19, 46, 52, 53]. While the number of willing participants was a mere handful, Chakraborty and Ghosh [52] is of the belief that such minority groups may motivate their community members, potentially increasing in the future. Hence, it is not unexpected that Karmakar et al. [53] reported that all potential body donors in their study affirmed that they would encourage family and friends to also consider body donation for the purpose of education.

#### 4.1.4 Correlation between socio-demographic factors and willingness to donate

Given that the willingness or reluctance to donate one's body is greatly influenced by socio-demographic factors [46], the microcosm of MBRU, represented by 322 members from 23 different nationalities and backgrounds, further enhanced the cultural and social diversity of the target population in this study. Furthermore, the diversity depicted in the results provided an opportunity to delve deeper into the population group who were willing to donate and to consider the existence of possible correlations between socio-demographic factors and willingness to donate one's body [54].

In the willingness to donate group, females were more willing to donate their bodies than males for the purposes of education and research. In the UAE, female individuals predominate the largest healthcare network (i.e., Abu Dhabi Health Services Company), possibly making them more knowledgeable about the value of body donation and hence are more willing to donate [55]. This finding was contrary to those of Saha et al. [19], Kostorrizos et al. [41], and Karmakar et al. [53], all of whom found that male individuals expressed a positive attitude towards willingness to donate. This attitude was attributed to males being more knowledgeable and having appropriate practice (i.e., seen donor pledge form, registered as donor) regarding body donation [53]. Conversely, female individuals have been noted to require some persuasion in this regard, with coping strategies of females highlighted as an influential factor behind this perception [53, 56].

In this study, willingness to donate once body was unaffected by the age of the participant. However, other studies reported an age-related pattern [47, 53, 57, 58]. According to Zhang et al. [47], individuals aged 18 and above are considered to be legal adults in China, which provides them with the capacity to legally sign, thereby encouraging them to pledge their bodies for the sake of education. While it was expected that the level of maturity would enable participants in the older group of our study to diplomatically weigh the advantages and disadvantages of body donation, lack of awareness may account for the low willingness to donate amongst this group.

Medical students benefit directly from whole body donation; hence it is not surprising that this group was most willing to donate. Despite similarity to the literature reviewed, willingness to donate by the student group was markedly higher in this study [19, 52, 59]. In addition, the higher educational level and/or age of faculty, which are likely to influence their overall maturity towards body donation, may explain why this group of participants represented approximately one-quarter of those willing to donate for educational and research purposes.

Since the majority of participants in this study were medical students, secondary education appeared to be the most prevalent educational qualification amongst the group willing to donate, differing greatly from the findings of Quiroga-Garza et al. [2]. This may owe to the impact of body donation on students' preclinical academic journeys. In fact, Zhang et al. [47] estimated that individuals with bachelor's degrees and/or postgraduate certification had a 50 % lower probability to donate their bodies than those without higher education certifications. Along with the statistically significant difference, this highlighted the association between educational level and body donation advocated by Oktem et al. [31].

As revealed by the socio-demographic distribution of this study, more than three-quarters of the participant population were of the Muslim religion, therefore religion was dichotomously divided into two groups, namely Muslim and Others. Likewise, the proportion of Muslim participants within the willingness to donate group was analogous to the socio-demographic distribution. However, this finding differed from that of Elamrani et al. [60] as the “Others” religious group was more willing to donate in their study. While these opposing views stem from religion and spirituality, they presumably underpin the rituals done as part of the last rites which most individuals would want to be performed on their bodies post-mortem [46]. Regardless of opinion or preference, past and present findings reinforce the fact that all religions support body donation for betterment of the world [19]. Moreover, it is worth noting that geographical location, race/ethnicity, societal norms, and cultural background also influence the value and belief systems of an individual, hence one's opinion about death and dying could be based on these factors which are likely to be misinterpreted as a religious barrier [6, 58]. On a similar note, filial piety, which is a central tenet of the less-known ancient belief system of Confucianism, emphasises the intactness of one's body at death [61, 62]. While the practice of Confucianism is still very much alive in East Asia, Jones and Nie [61] built an argument against Confucians' reluctance to donate by basing it upon Ren, the foundational virtue of Confucianism, which denotes benevolence and altruism, thus allowing for the donation of one's body. In view of the aforementioned societal, cultural, racial/ethnic and geographical factors that stand against willingness to donate, Mazyala et al. [63] proposed that these can be addressed by increasing awareness among anatomy faculty and the general public.

Given that statistically significant differences were noted for gender, role of participant, level of education, and religion in this study, it was understood that these socio-demographic characteristics act as the motivating factors behind body donation for educational and research purposes.

#### 4.1.5 Participants’ perceptions: body donation programs, fate, and role of donated body

In many parts of the world, Anatomy departments depend largely on body donation programs as the main source of teaching material [41, 64]. While willed body donation programs are well-established and recognised in North America, Europe, and China; those in India are still in their stages of infancy [23, 53, 65]. Nevertheless, as the number of medical schools continue to increase in developing and developed countries, there is an ardent need to establish institutional body donation programs [66]. The successful establishment of such programs hinges on the relevant socio-demographic characteristics, but also require prior consideration of the number of body donation programs within the region, fate of the dead body after use in education and research, as well as the students’ perceived role of the dead body [2, 6]. In fact, half of the participant population in the present study were aware of the fact that no in-house body donation programs exist in the UAE, with MBRU procuring whole-body donors from a federally approved American-based non-profit research organization, accredited by the American Association of Tissue Banks [67]. For the remaining half of the participant population, the selected number of existent body donor programs in the UAE were distributed among the other available options. As Erdogan et al. [68] stated that there is paucity in published information pertaining to body donation programs, virtual and written press releases may be useful in educating the participants that comprise the latter half, thereby promoting the development of such programs.

With regard to the journey of body donation in a medical school, it generally commences with the procurement of the donated body from a licensed source, followed by preservation of the body, then use in anatomical education and research, and eventually concludes with the respectful disposal of the remains of the donated body [69]. While the junctures of this journey differ, continuous and consistent adherence to ethical guidelines is paramount [69]. Moreover, Riederer et al. [21] and Ghosh [70] advocated that the ethical disposal of the body post-dissection, either through cremation or burial, should emulate one of immense respect and honour. Only one-third of participants in this study were aware of this recommendation, with the majority citing that they were not aware of the fate of the dissected body. In fact, the perception of the former group was in line with the practice of ethical disposal undertaken by MBRU. As per the contract with the licenced supplier, donated bodies at MBRU are retained for a period of five years from the date of procurement. Upon expiry of the five-year contract and consultation with the local authorities, the body (dissected or whole) is handed over to the Public Health Services Department of the local municipality for subsequent performance of the last rites and burial. Documentation pertaining to the respectful disposal are then provided to the licensed supplier. With these aspects of ethical practice at the forefront, Ghosh [69] recommended that the ethical principles emanating from the dissection hall experience provide medical students with a simulacrum of the professional code of conduct that they will uphold in clinical practice.

Indeed, the role of the dead body is perceived quite uniquely in different cultures, geographical locations, and educational institutes. In Thailand for instance, “Ajarn yai”, which translates to “Great Teacher” in the Thai language, is a noble status given to all donated bodies [71]. It demonstrates utmost respect to teachers which may be unfamiliar in western cultures. Interestingly, by providing this honourable status and title to donated bodies, the Thai population were incentivized to pledge their bodies for post-mortem donation [71]. Although medical students at MBRU are advised to treat the dead body with absolute care and respect, a manner in which doctors would care for their patients, approximately two-thirds of the study population expressed that they preferred to perceive the dead body as the “First Teacher” from whom a medical student learns. This view also resonated with those reported in previous studies [46, 64, 72]. According to Bohl et al. [72], maintaining this view has proven most effective in fostering students’ emotional development. As a matter of fact, an Indian medical school created a first-year module, entitled “Cadaver as a First Teacher”, to disseminate the humanistic and ethical values associated with body dissection [46, 64]. Therefore, application of the Thai perception may resolve the ethical dilemma that surrounds body donation and may encourage the general population to participate in this altruistic act.

### 4.2 Cluster analysis

As revealed by the cluster analysis, of the 44 (29.5 %) participants who expressed their willingness to donate their bodies for the purpose of education and research, only 28 (20.6 %) participants in the total study population will potentially donate their bodies. The selected attributes, according to which cluster membership was based, included gender, role of participant at the university, religion, and level of education – the latter two of which were similar to the attributes selected by Gürses et al. [34]. In addition, the predominance of secondary and higher/tertiary levels of education in the reluctant and donor groups, respectively, differed from the educational levels predominating clusters 1 and 2 in the study of Gürses et al. [34]. Since cluster analyses provide a means to configure the potential donor group, recruitment strategies of body donation programs can be tailored to focus on this particular group, thereby providing an effective means of increasing anatomical donations for education [33].

### 4.3 Qualitative analysis

According to the sociological study by Hafferty [73], which described the behaviour of students in the dissecting hall, students are generally categorised into two groups, namely those empathetic individuals who treat the dead body as a living being, and those who are emotionally detached individuals who view the dead body as a biological specimen [6, 73]. When investigating the opinion about body donation and the impact of it on a potential donor's life – particularly a donor with a medical background; it is necessary to be culturally-sensitive to the existence of the above-mentioned two groups. Regrettably, the applicability of these behavioural categories amongst non-medical donors of the general public is yet to be determined [6].

In this study, participants were provided with the opportunity to express their opinion about impact (direct/indirect) of body donation on their lives and/or in the educational setting, with Resource, Humanitarian, Barrier, and Awareness emerging as the focal areas of the thematic framework. These themes indicated that participants’ perceptions were indeed manifold [65].

#### 4.3.1 Resource

The theme Resource, which was predominantly expressed by participants, revealed the intangible value of body donation in medical education and research, thereby corroborating the literature reviewed [74, 75]. Participants’ responses also indicated that the sub-themes of educational tool, advancement of science, and knowledge translation embodied the entirety of the main theme. Souza et al. [64] articulated that the dead body assumes the form of a learning tool that offers a fascinating learning process in which the “dead” teach the “living”. The perceptions of many participants in the present study resonated with this as they stated their preference to learn human anatomy through dissection rather than through classical textbooks and plastic models because it provided an unforgettable hands-on experience. The opportunity to foster teamwork and collaborative learning was also previously identified [64]. Moreover, participants appreciated the realism afforded by this learning process as they explained how the lecture content directly translates to what is seen in the dead body. Veritably, dissection of dead bodies is reported to be the most acceptable way of learning anatomy [76, 77]. To a lesser extent, participants mentioned the contribution to medical science research, a notion also conveyed by Jiang et al. [65]. Hence, it is not surprising that the contribution to medical education acts as primary motivating factor for many potential donors [78].

#### 4.3.2 Humanitarian

In line with earlier studies [6, 65], this theme portrayed body donation as an altruistic act and appeared as a key motivator. More specifically, participants' quotes that were representative of the sub-themes (i.e., respect and honour, and altruism) all conveyed a sense of gratitude – endorsing the results of Chang et al. [79]. Feelings of respect and honour were expressed for the sacrifice made by a once-living individual for the advancement of education and medical research, which many participants referred to as the self-less act of giving (i.e., altruism). Hence, the above-mentioned sub-themes were mutually inclusive to the main theme of Humanitarian in the present study. Additionally, Souza et al. [64] found that respect may also be associated with the way one handles the dead body during dissection that later translates to the way one respects a patient in clinical practice, however this was not highlighted by participants’ perceptions in this study. Interestingly, one participant referred to the dead body as “someone”, alluding to empathy and adding an element of humanism to a process that is frown up by so many. In fact, a similar humanistic view, which was shared by a participant in a previous study, revealed the personal attachment that came with being responsible for careful handling of the assigned body, as well as the unsaid responsibility to utilize this unique experience (made possible by the donated body) to become a competent doctor [79].

#### 4.3.3 Barrier

A minority group of participants communicated responses that were representative of the “Barrier” theme as they differed greatly from those that connotated the positive themes of the framework (i.e., Resource, and Humanitarian). When reviewed further, the range of responses reflected the barrier-like components of religion, family, trigger of bereavement, ethical concerns, socio-cultural factors and technological alternatives. In line with similar studies conducted in the ME, the difference in religious and cultural backdrops between the region and the western world is deemed to shape participants' attitudes [54, 65]. Of course, end-of-life planning often requires consultation with one's family, hence they have been reported to also sway the decision-making process of the individual [80]. Conversely, Saha et al. [19] communicated that family members were one of the main sources responsible for the motivation behind participants' willingness to donate. Few participants found body donation to be completely unethical, especially with the recent availability of technological alternatives. Given participants' personal experiences in the dissection room, ethical concerns may stem from a fear of disrespect. Some participants described body dissection to be quite an uncomfortable and traumatic experience as it reminded them of the death and loss of a loved one. Such intimate and personal emotional experiences are not unknown [79]. In fact, Chang et al. [79] observed that as participants progressed through dissection in their anatomy courses, positive emotions diminished. This non-empathetic state was further exasperated by regular assessments and the intense workload [79]. According to Dosani and Neuberger [81], this emotional state can be addressed at the initial stages by showing participants the movie “Anatomy and Humanity” prior to the first exposure to dissection as this approach has proven to reduce negative emotions. In such instances, it is also crucial to understand the reason behind this perception and perhaps evaluate the emotional climate of the dissection room [6].

#### 4.3.4 Awareness

With regard to the awareness about body donation for the purpose of teaching and research, this theme shed light on aspects related to the lack of it, need for it and the change in perception post-exposure. As medical students at MBRU were the ones who received their anatomy education mainly through body dissection, they appeared to be the majority group who found that body donation impacted their lives, and in some cases to the extent that they stated they were willing to donate their bodies for educational purposes. It is also likely that this impact fostered a new-found passion to pursue a career in anatomically oriented fields such as pathology, radiology, and surgery [54]. Additionally, the lack of awareness, which was alluded to through participants’ responses, emphasized the need for well-established outreach programs that educate the student about this subject. It may prove beneficial to hold a commemoration ceremony in the presence of leaders from the various religious and cultural backgrounds, which will provide a more realistic interpretation of death, dying and altruism and eliminate uncertainty [2].

### 4.4 Strengths

In attempt to minimize bias, non-medical vocabulary was used in the construction of the questionnaire. As the questionnaire was self-administered, interviewer bias was further reduced. Moreover, the English version of the questionnaire was pilot tested to ensure the questions were organized in a logical order, thus allowing for ease of comprehension. Although this study was limited to a specific population group within one university, it conveyed unique perceptions that were possibly reflective of diverse socio-cultural differences as the participating group consisted of individuals from 23 different nationalities. This was also the first study of its nature in the MENA region (ME and North Africa) to assess the perceptions of faculty, staff, and students regarding body donation for educational and/or research purposes. The data reported in this study may supplement the paucity of literature regarding this particular subject in the ME.

### 4.5 Limitations

Approximately 50 % of the initial target population completed the questionnaire, which may owe to the fact that this study was conducted within a limited time frame as a component of the students' research project course. The questionnaire was also amongst many others shared with all faculty, staff, and students via a single electronic link on the institution's communications and external relations platform. Many individuals within the target population may have opted to refrain from participating in the study due to the sensitive nature of subject in question.

### 4.6 Future research

It is recommended that future studies incorporate a larger sample size to compare the perceptions and attitudes of medical students towards body donation pre- and post-exposure to dissection. Furthermore, an individual's academic performance in anatomy courses may be correlated with their willingness or reluctance to donate for additional interpretation.

## 5 Conclusion

This study not only assessed the level of awareness of faculty, students, and staff regarding body donation for teaching and research purposes, but also educated participants about this controversial topic. Although only 44 (29.3 %) of participants expressed willingness to donate their bodies for educational and research purposes, a majority of 111 (74.0 %) participants considered body donation to be appropriate. A potential donor group was also identified within the target population group through cluster analysis. Interestingly, the number of potential donors 28 (20.6 %) represented almost two-thirds of participants within the willingness to donate group 44 (29.5 %).

On the contrary, reluctance to donate 106 (70.7 %) one's body was influenced by religion, following by a psychological barrier, and familial reasons. In view of the statistically significant differences yielded for gender, role of participant, level of education, and religion; these socio-demographic characteristics appeared to be the motivating factors behind body donation for educational and research purposes. Furthermore, the focal areas emerging from the thematic framework (i.e., Resource, Humanitarian, Barrier, and Awareness) were reflective of the diverse perceptions of participants.

The above-mentioned findings may provide a means of revisiting recruitment strategies when body donation programs are being established. They will also assist to spread further awareness about the value of body donation for research and educational purposes, thereby eliminating the stigma attached to body donation and positively influencing societal perceptions.

## Declarations

### Author contribution Statement

Nerissa Naidoo:Conceived and designed the experiments; Analyzed and interpreted the data; Wrote the paper.

Ghadah A. Al-Sharif: Conceived and designed the experiments; Performed the experiments.

Raeesa Khan: Analyzed and interpreted the data; Wrote the paper.

Aida Azar, Amar Omer: Analyzed and interpreted the data.

### Funding statement

This research did not receive any specific grant from funding agencies in the public, commercial, or not-for-profit sectors.

### Data availability statement

Data will be made available on request.

### Declaration of interests statement

The authors declare no conflict of interest.

### Additional information

No additional information is available for this paper.
